# Assessment of Retinal Microangiopathy in Patients with Balkan Endemic Nephropathy Using Optical Coherence Tomography Angiography—A Pilot Study

**DOI:** 10.3390/medicina60010192

**Published:** 2024-01-22

**Authors:** Jasmina Djordjevic-Jocic, Jovana Cukuranovic Kokoris, Branka Mitic, Dragan Bogdanovic, Marija Trenkic, Nevena Zlatanovic, Hristina Jocic, Rade Cukuranovic

**Affiliations:** 1 Clinic of Ophthalmology, Faculty of Medicine, University of Nis, Blvd. Dr Zorana Djindjica 81, 18000 Nis, Serbia; marija.trenkic@gmail.com; 2 Department of Anatomy, Faculty of Medicine, University of Nis, 18000 Nis, Serbia; jovana.cukuranovic.kokoris@medfak.ni.ac.rs (J.C.K.); radecukuranovic@yahoo.com (R.C.); 3 Clinic of Nephrology, Faculty of Medicine, University of Nis, 18000 Nis, Serbia; dmmitic@ptt.rs; 4 Department of Statistics, State University of Novi Pazar, 36300 Novi Pazar, Serbia; draganbogdanovic@gmail.com; 5 Health Center Nis, 18000 Nis, Serbia; drnevenazlatanovic@gmail.com; 6 Clinic of Neurosurgery, University Clinical Center of Nis, 18000 Nis, Serbia; tina.jocic@gmail.com

**Keywords:** optical coherence tomography angiography, microangiopathy, retina, kidney

## Abstract

*Background and Objectives*: It is well known that alterations in microvascular structure and function contribute to the development of ocular, renal, and cardiovascular diseases. Accordingly, the presence of fundus vascular changes in patients suffering from chronic kidney disease (CKD) and Balkan endemic nephropathy (BEN) may provide information of prognostic value regarding the progression of renal disease. This study aimed to examine the associations between clinical characteristics and retinal optical coherence tomography angiography (OCTA) parameters in patients with BEN and compare them with those in CKD. *Materials and Methods*: This pilot study, conducted from March 2021 to April 2022, included 63 patients who were divided into two groups: the first group consisted of 29 patients suffering from BEN, and the second was a control group of 34 patients with CKD. Demographic, laboratory, clinical, and medication data were noted for all the patients included in this study. Each eye underwent OCT angiography, and the results were interpreted in accordance with the practical guide for the interpretation of OCTA findings. *Results*: Statistically significantly higher levels of total serum protein and triglycerides were recorded in the BEN group than in the CKD group, while the level of HDL cholesterol was lower. Based on the performed urinalysis, statistically significantly higher values of total protein and creatinine were detected in patients with CKD compared to the BEN group. It was demonstrated that the OCTA vascular plexus density of certain parts of the retina was in significant association with systolic and diastolic blood pressure, creatinine clearance, urinary creatinine, total cholesterol, diabetes mellitus type 2, age, body mass index, total serum and urinary protein, sCRP, and diuretic and antihypertensive treatment. *Conclusions*: In comparison with CKD, BEN leads to more significant disturbances in retinal vasculature density.

## 1. Introduction

Chronic kidney disease is a progressive condition characterized by structural and functional changes in the kidneys that are present for at least 3 months, regardless of the cause [1,2]. It has been estimated that between 8% and 16% of the population worldwide may be affected by CKD. This disease is less prevalent in high-income countries than in low-and middle-income countries, and is often underrecognized by patients and clinicians [3]. The global increase in CKD is due to the growth of the elderly population as well as the presence of other chronic pathological entities, such as cardiovascular diseases, diabetes, and cancer [4,5,6]. Balkan endemic nephropathy (BEN) is a familial tubulointerstitial disease with an insidious onset in the fifth decade and gradual progression to terminal renal failure [7,8]. Hall et al. were the first to indicate tubular proteinuria as an early sign of endemic nephropathy [9].

To date, the link between ocular and renal health has been clearly documented in numerous studies. Specifically, it has been shown that age-related macular degeneration (AMD) and CKD share many risk factors, such as hypertension, diabetes, and smoking. Hypertension, because of its role in kidney disease and possibly in the development of choroidal atherosclerosis leading to AMD, represents a particularly interesting common risk factor [10]. However, in the early 2000s, it was shown that, regardless of age, hypertension, diabetes, and other risk factors, there is a strong association between the characteristics of retinopathy and renal dysfunction [11]. This can be partially explained by the fact that basement membrane thickening represents a common pathogenic mechanism for retinopathy and nephropathy [12]. In subsequent studies, associations between kidney disease and AMD, as well as AMD and kidney function, have also been confirmed [13].

Previous studies, although not so numerous, have shown that in patients with CKD and BEN, there is disintegration at the level of vascular arcades in the foveal avascular zone (F.A.Z.), expansion of the F.A.Z., the parafovea zone, as well as reduced capillary density. Interestingly, changes in the capillary network at the level of the macula also exist in patients with CKD and BEN who do not have clinically visible AMD. Due to new treatment options, OCTA can be very important in the early detection of AMD and for monitoring patients with CKD and BEN.

The aim of this study was to examine the associations between clinical characteristics and retinal OCTA parameters in patients with BEN and compare them with those in patients with CKD.

## 2. Materials and Methods

### 2.1. Patient Population

This monocentric pilot study included 63 patients (29 women and 34 men) who visited the outpatient department of the Clinic for Nephrology and Hemodialysis of the University Clinical Center Nis, Serbia, from the beginning of March 2020 to the end of April 2021. The patients were divided into two groups: the first group consisted of 29 patients suffering from BEN. The second was a control group of 34 patients with CKD.

Demographic, laboratory, clinical, and medication data for all the patients in this study were obtained from interviews and the relevant medical records (patient medical histories and electronic databases).

The inclusion criteria were patients of both sexes, male or female subjects ≥40 years of age with a diagnosis of CKD or BEN established according to the current guidelines [14,15]. Due to the small number of patients with BEN in the general population, those with type 2 diabetes were also included in this study.

The exclusion criteria were a history of recurrent fever or clinical and laboratory signs of sepsis; severe acute or exacerbation of chronic systemic disease; any malignancy; proteinuria >10 g/day and/or serum albumin <20 g/L; history of congestive heart failure or stroke within the previous three months; or moderate liver function test abnormalities >1.5 times normal upper limit values.

A written informed consent was obtained from all the patients before their inclusion in this study. This research was conducted according to the ethical guidelines of the 1975 Helsinki Declaration and approved by the Ethics Committee of the Faculty of Medicine, University of Niš, Niš, Serbia.

All the patients in this study underwent a physical examination that assessed their vital parameters and measured their height/weight to determine their body mass index (BMI). The blood samples used for the biochemical measurements were obtained after an overnight fast of at least 10 h. The glomerular filtration rate (eGFR) was estimated according to the modification of diet in renal disease (MDRD) formula. Urine samples were collected for the measurement of microalbuminuria and creatinine levels.

### 2.2. Ophthalmological Examinations

All patients underwent a routine ophthalmological examination at the Ophthalmology Clinic of the University Clinical Center Niš during the research period.

Clinical ophthalmological examination and OCTA were performed by two specialist ophthalmologists at the Ophthalmology Clinic of the Niš University Clinical Center. As part of the clinical ophthalmological examination, slit lamp biomicroscopy of the anterior segment, slit lamp gonioscopy, and direct slit lamp Goldmann three-mirror gonioscopy of the anterior chamber angle were performed.

Simultaneous color stereo fundus photography centered on the optic disk (30°) was achieved using a Nidek 3D×/NM camera (Nidek Technologies, Inc., Pasadena, CA, USA).

Each eye underwent OCT angiography (RTVue XR Avanti System with AngioVue software version 2017.1; Optovue, Fremont, CA, USA). All the measurements were performed automatically. The results obtained through these measurements are reproducible and referenced, as shown by numerous studies. The obtained results of the performed OCTA analysis were interpreted in accordance with the practical guide for the interpretation of OCTA findings presented below [16].

The OCTA parameters monitored in this study were based on an ETDRS map and a section size of 3 × 3 mm. Accordingly, the macula was divided into two or three regions: the central F.A.Z. region, the perifoveal region, and the parafoveal region. Subsequently, the map was divided into the superior, inferior, temporal, and nasal region and into two hemispheres: superior and inferior.

The F.A.Z. is a zone that represents the area of the macula without a capillary network. The size of the F.A.Z. was defined on the OCTA RTVue Avanti System through the following three parameters: F.A.Z., in mm^2^, F.A.Z., perimeter in mm, and capillary network density. Foveal density (F.D.—a 300-micron-wide ring that surrounds the F.A.Z.) was obtained as the ratio of the total number of pixels of the capillary network to the total number of pixels, multiplied by 100, and expressed as a percentage. The acquired images were checked for their quality (signal strength of more than 6/10) as well as for the absence of artifacts by two independent ophthalmologists. Poor-quality images with either significant motion artifacts or extensive incorrect segmentation were excluded and repeated.

The boundary between the layers was also generated automatically. Blood vessel density and flow index were analyzed in a radius of 1.25 mm from the fovea, excluding a zone of 0.3 mm.

The parameters monitored in all three sections were as follows: percentage—marked with the standard symbol %, which represents the ratio between signal-positive pixels in relation to the total result of pixels in the observed region and the F.A.Z. area parameter expressed as mm^2^, which was analyzed for the S.V.D. and DVD pixels.

### 2.3. Statistical Analysis

The obtained data were analyzed using “R” version 2.2.1 (R Foundation for Statistical Computing, Vienna, Austria), a software language for statistical computing. Continuous variables were shown as the mean with standard deviation or median with interquartile range, depending on the normality of the distribution. Absolute and relative numbers present categorical variables. To compare the values of continuous variables between two groups, the Student’s *t*-test was used for normally distributed data, and the Mann–Whitney U test was employed for non-normally distributed data. Pearson’s Chi-square test and Fisher’s exact test were used to compare categorical variables between groups. Multivariate regression models using the backward conditional method were performed to estimate associations between candidate independent variables and dependent variables. The level of significance was set at *p* < 0.05.

## 3. Results

Table 1 provides the baseline characteristics and the clinical variables of patients with BEN and CKD. This study included 63 patients, 29 patients (12 females and 17 males) suffering from BEN and 34 patients (17 females and 17 males) with CKD, with an average age of 66.90 ± 10.3 and 70.22 ± 15.33, respectively. There was no statistically significant difference between the groups in terms of their mean age or gender. However, there were significantly more smokers in the BEN group (*p* = 0.002), as well as patients with prescribed antihypertensive therapy, compared to the CKD group (*p* = 0.034). Also, blood analyses showed statistically significantly higher levels of total serum protein (*p* < 0.001) and triglycerides (*p* = 0.008) in the BEN group than in the CKD group. In contrast, their HDL cholesterol level was lower (*p* < 0.001). Based on the performed urinalysis, statistically significantly higher values of total protein (*p* < 0.001) and creatinine (*p* < 0.001) were detected in patients with CKD compared to the BEN group (Table 1).

The results of the OCTA examination showed statistically significantly higher values of vascular plexus density in the whole image of both the superficial and deep layers of the retina, while in certain sectors of the retina, this significance was also recorded in the superior and inferior hemisphere, fovea, parafovea, temporal, superior, and nasal quadrant of the superficial layer, and the superior and inferior hemisphere, fovea, parafovea, temporal, and nasal quadrant of the deep layer of the retina in patients with CKD compared to the BEN group. However, there was no statistically significant difference in the thickness of the superficial and deep retinal layers between the compared patient groups in the whole image and in relation to specific sectors (Table 2).

Multivariate regression models using the enter method were performed to assess the associations between dependent variables. The values of the regression coefficients and their 95% CIs were calculated. The associations of OCTA parameters were statistically significant with the standard clinical, demographic, and biochemical markers in patients with BEN and CKD, as shown in Table 3 and Table 4. It was demonstrated that the vascular plexus density (VPD) of the deep whole image, deep superior quadrant, and deep nasal quadrant in patients with BEN and CFT and the deep fovea in CKD patients were in significant association with diastolic blood pressure. A significant interrelation was also established between creatinine clearance and the VPD of the deep superior hemisphere in patients with BEN and the deep fovea, superficial parafovea, superficial superior, and nasal quadrant in CKD patients. Likewise, the VPD of the deep superficial hemisphere and the deep superior quadrant in BEN patients and the F.A.Z. and superficial whole image in CKD patients were in significant association with urinary creatinine. Finally, total cholesterol, systolic blood pressure, diabetes mellitus type 2, age, BMI, total serum and urinary protein, sCRP, and diuretic and antihypertensive treatment were also shown to be independently associated with the vascular plexus density of certain parts of the retina in examined populations (Table 3 and Table 4).

## 4. Discussion

Previous studies have shown that OCTA can detect changes in the macular capillary network of patients with diabetes mellitus [17], arterial hypertension [18], and chronic kidney disease [19]. Therefore, the interest of researchers dealing with this topic has shifted towards the early detection of circulatory alterations, which would impact the timely prevention of complications on the retinal vascular network.

The vasculature system of the retina consists of the central retinal artery, which supplies the inner retina, and the choriocapillaris, which supplies the retinal pigment epithelium and outer retina (primarily composed of photoreceptors) [20]. The blood supply of the F.A.Z.is also derived from the choriocapillaris [21]. In mammals, the photoreceptor layer is entirely free of blood vessels and is supplied with oxygen by diffusion from adjacent vascular structures. The contribution of oxygen to visual function depends on the light and dark conditions; in the light, all O_2_ comes from the choroidal circulation, while in the dark, O_2_ diffuses from both the choroid and the retinal circulation. Due to the outer retina’s avascular nature and photoreceptors’ high oxygen demand, this region is at risk of hypoxia, especially after dark adaptation [22].

The thickness of the choroid plexus decreases with age, but microvascular changes also occur in the retinal circulation, which is mainly susceptible to arteriosclerosis and accompanied by decreased capillary density in the fovea [23,24]. As the photoreceptors are supplied with blood via the choroidal vasculature, the reduction in choroidal thickness results in their damage, representing the predisposing factor for vision loss.

Dyslipidemia is one of the critical risk factors in the development of atherosclerosis that leads to vessel wall responses, including endothelial dysfunction, smooth muscle cell proliferation, lipid accumulation, foam cell formation, and, eventually, necrosis and plaque development [25]. In terms of ophthalmological pathology, dyslipidemia is involved in the pathogenesis of dry AMD through the accumulation of oxidized lipids, lipid-associated molecules, and inflammatory debris, which precedes drusen formation. Consequently, the transport of nutrients and waste between the RPE cells and the choroidal vessels is slowed down, leading to disturbances in RPE cell function [26]. In our study, the imaging analysis results showed statistically significantly lower values of retinal vessel density in both the superficial and deep layers in patients with BEN compared to the CKD group, which could be potentially associated with hyperlipidemia. The relationship between hyperlipidemia and reduced capillary density indexes was also demonstrated in patients with type 2 diabetes mellitus using a swept light source OCTA device [27].

On the other hand, it is well known that the environmental phytotoxin aristolochic acid (A.A.), contained in *Aristolochia clematitis*, represents the causative agent of BEN. Regarding aristolochic acid intoxication, some reports suggest the involvement of the organic anion (O.A.) transporter (O.A.T.) family in AA-mediated nephrotoxicity. The O.A.T. family comprises over 10 transmembrane proteins and belongs to the solute carrier 22 (SLC22) subfamily. The O.A.T.s are distributed in almost all barrier epithelia of the body, as well as in the endothelium. Moreover, it has been reported that these transporters are also expressed in the retina, which could explain the significantly reduced capillary density in patients with BEN compared to those with CKD [28].

In addition, a recent study showed that blood pressure and eGFR can influence retinal capillary density and should be considered for studies characterizing eye diseases in human populations. Additionally, this study also showed that changes in capillary density in hypertensive patients were found exclusively in the deep retinal layer, suggesting that systemic hypertension may affect one retinal vascular layer differently than another [29]. Likewise, in patients with systemic hypertension, in whom the relationships between quantitative OCTA parameters of the retinal capillary network and myocardial abnormalities documented on magnetic resonance imaging of the cardiovascular system were examined, a lower superficial capillary density was found in the group with myocardial fibrosis compared to subjects without myocardial structural changes [30]. On the other hand, other systemic diseases, such as diabetes, can show widespread changes in both vascular plexuses [27].

The independent factors that were shown to be independently associated with the vascular plexus density of specific parts of the retina in our study were smoking, BMI, type 2 diabetes mellitus, and sCRP, diuretic, and antihypertensive treatment. Although the results of the conducted studies are still controversial, there are data on the negative impact of smoking on the vascular plexus density in the retina. It was noted that the total vascular density, parafoveal vascular density, and perifoveal vascular density in the deep capillary plexus observed with OCTA were significantly lower in the smoking group and may even occur under a low pack-year exposure [31]. Additionally, it was demonstrated that cigarette smoking represents an independent risk factor for reduced density of the deep retinal capillary plexus [32,33]. An in vitro study showed that chronic nicotine exposure elevates the resting intracellular calcium ion concentration and upregulates the expression of the canonical transient receptor potential channels in cultured vascular smooth muscle cells. These cells surround the arteries and arterioles in the retina, while the capillaries are not entirely covered by pericytes and their processes, indicating that the main cells controlling the vascular tone differ in the parafoveal and peripapillary regions. Therefore, the difference in the degree of contraction of blood vessels in the various regions may explain the changes in vascular density in smokers [34]. Also, smoking has been reported to affect the vascular endothelium by increasing oxidative stress, decreasing antioxidant vitamin C levels, and causing abnormal nitric oxide activity [35]. On the other hand, it is well known that cadmium, a natural element and a toxic metal found in cigarettes, exhibits nephrotoxic properties [36]. Finally, the potential role of smoking as a risk factor for the development of BEN has also been described [37].

As for the BMI, overweight and obesity have been shown to be associated with retinal microvasculature abnormalities regardless of the presence of diabetes and hypertension at all ages [38,39,40,41]. Compared to the healthy controls, the densities of the fovea superficial capillary plexus, average deep capillary plexus, fovea deep capillary plexus, parafovea deep capillary plexus, and perifovea deep capillary plexus in patients with metabolic syndrome, as well as the fovea superficial and deep capillary plexus in metabolically healthy obesity patients, were significantly lower [40]. In children, newly developed obesity was associated with lower vessel density in the inferior parafovea, nasal parafovea, and temporal perifovea of the deep vascular complex, higher vessel density in the fovea of the superficial and deep vascular complexes, and a smaller foveal avascular zone in comparison with controls [38]. Regarding kidney disease, a high BMI represents one of the most substantial risk factors for new-onset CKD. In obese patients, compensatory hyperfiltration occurs to meet the heightened metabolic demands of the increased body weight.

Consequently, an increased intraglomerular pressure can lead to kidney damage and raise the risk of developing CKD in the long term [42]. Diabetes mellitus type 2, as a chronic metabolic disorder characterized by persistent hyperglycemia, predominantly affects the microvascular circulation of the retina and kidney through various mechanisms, including oxidative stress and inflammation [43]. It has been shown that vascular density in OCTA correlates significantly and linearly with disease severity in diabetic retinopathy. Furthermore, the parafoveal vascular density of the superficial capillary plexus and the foveal vascular density of the choriocapillaris were introduced as biomarkers in the prediction of vision loss in patients with diabetes [44]. Finally, concerning CRP, an acute inflammatory protein that increases in response to inflammation, numerous studies have shown that retinal vessel density and retinal perfusion reflect, in addition to systemic hemodynamic changes, a systemic inflammatory response [45].

### Study Limitations

A limitation of this study was the small number of patients in both examined populations. Additionally, due to the difficulty of including patients with BEN, which is a rare disease, patients with coexisting type 2 diabetes were not excluded from this study, which may affect the results obtained from our research. Finally, the automated, programmed algorithm in the AngioVue system only segments two retinal capillary plexuses: the superficial capillary plexus and the deep capillary plexus, while the middle capillary plexus that is defined by swept-source OCT could not be calculated.

## 5. Conclusions

Since patients with BEN were shown to have more significant disorders of retinal vasculature density compared to those with CKD, detecting capillary plexus abnormalities by OCTA at a very early stage, which are often clinically indistinguishable, allows for a timely and adequate treatment and the prevention of potential complications, such as vision loss. Currently, there are effective treatment methods for tissue neovascularization (e.g., anti-VEGF or retinal laser photocoagulation), but no treatment method affects retinal tissue ischemia.

## Figures and Tables

**Table 1:** The demographic, laboratory, clinical, and medication data and OCTA parameters of the patients included in this study.

Variable	BEN (*n* = 29)	CKD (*n* = 34)	*p*-Value
Gender (number (%))			
Males	17 (58.62%)	17 (50.00%)	0.494
Females	12 (41.38%)	17 (50.00%)	
Age	66.90 ± 10.3	70.94 ± 9.44	0.112
Body mass index (kg/m^2^)	28.86 ± 3.74	26.88 ± 5.29	0.089
Diabetes mellitus type 2 (number (%))	7 (24.14%)	6 (17.65%)	0.526
Hypertension (number (%))	27 (93.10%)	30 (88.23%)	0.507
Antihypertensive therapy (number (%))	27 (93.10%)	25 (73.53%)	0.034
Diuretic therapy (number (%))	15 (51.72%)	12 (35.29%)	0.188
Smokers (number (%))	6 (20.69%)	0 (0.00%)	0.002
Systolic blood pressure (mm Hg)	140.52 ± 22.53	135.59 ± 19.06	0.357
Diastolic blood pressure (mm Hg)	84.83 ± 14.05	85.59 ± 14.4	0.833
Serum creatinine (μmol/L)	186.97 ± 60.88	206.25 ± 24.90	0.190
Creatinine clearance (mL/min)	49.49 ± 18.44	49.44 ± 29.35	0.994
Total serum protein (mmol/L)	74.88 ± 5.37	65.35 ± 7.21	<0.001
Serum albumin	41.53 ± 4.73	39.84 ± 6.53	
Total cholesterol (mmol/L)	5.11 ± 0.91	5.45 ± 1.18	0.198
HDL cholesterol (mmol/L)	1.08 ± 0.27	1.50 ± 0.34	<0.001
LDL cholesterol (mmol/L)	3.00 ± 0.70	3.35 ± 1.06	0.132
Triglycerides (mmol/L)	2.75 ± 2.54	1.39 ± 0.55	0.008
Serum glucose (mmol/L)	5.65 ± 1.17	5.85 ± 0.67	0.423
Urinary albumin (mg/L)	18.68 (2.84–634.57)	22.10 (2.31–817.36)	0.733
Total urinary protein (mg/L)	174 (0.72–1470)	245 (0.56–1524)	<0.001
Urinary creatinine (mmol/L)	11.45 (3.52–18.35)	17.13.(8.45–27.03)	<0.001
Urinary albumin creatinine ratio (mg/mmol)			
Urinary protein creatinine ratio (mg/mmol)			

Values are given as a number (percentage), mean (standard deviation), and median (5th–95th percentile). *p* < 0.05 indicates statistical significance.

**Table 2:** OCTA parameters—zones and sectors of the retina by layers.

Variable	BEN (*n* = 29)	CKD (*n* = 34)	*p*-Value
Central foveal thickness (CFT)	267.86 ± 46.85	266.29 ± 43.93	0.892
FAZs	0.30 ± 0.09	0.29 ± 0.09	0.786
FDs	45.98 ± 6.14	46.19 ± 6.19	0.896
Superficial layer of the retina—vascular plexus density			
Whole image	40.11 ± 8.75	47.34 ± 5.24	<0.001
Superior hemisphere	41.90 ± 5.98	49.1 ± 5.93	<0.001
Inferior hemisphere	41.18 ± 5.64	45.47 ± 6.19	0.006
Fovea	23.38 ± 6.52	33.98 ± 8.79	0.034
Parafovea	44.08 ± 6.23	49.04 ± 6.19	0.002
Temporal quadrant	42.62 ± 6.90	47.85 ± 7.75	0.006
Superior quadrant	45.10 ± 6.77	50.15 ± 6.94	0.005
Nasal quadrant	43.60 ± 6.35	50.27 ± 6.67	<0.001
Inferior quadrant	45.03 ± 6.80	48.17 ± 6.23	0.063
Superficial layer of the retina—thickness by sector			
Whole image	261.36 ± 23.21	264.5 ± 20.77	0.573
Superior hemisphere	313.77 ± 20.33	314.35 ± 17.81	0.904
Inferior hemisphere	294.52 ± 59.61	291.27 ± 72.7	0.848
Fovea	277.1 ± 37.76	273.53 ± 33.91	0.694
Parafovea	295.52 ± 21.01	299.77 ± 19.54	0.409
Temporal quadrant	317.32 ± 30.11	316 ± 27.96	0.857
Superior quadrant	317.84 ± 26.5	318.44 ± 23.37	0.924
Nasal quadrant	294.19 ± 45.67	301.65 ± 41.47	0.499
Inferior quadrant	285.61 ± 38.16	285.59 ± 39.26	0.998
Deep layer of the retina—vascular plexus density			
Whole image	47.37 ± 5.41	40.7 ± 8.36	<0.001
Superior hemisphere	48.49 ± 6.04	42.12 ± 5.9	<0.001
Inferior hemisphere	45.27 ± 6.42	41.71 ± 5.56	0.023
Fovea	32.26 ± 7.13	23.17 ± 22.81	0.034
Parafovea	49.51 ± 6.35	44.46 ± 5.99	0.002
Temporal quadrant	48.01 ± 5.69	43.06 ± 6.59	0.002
Superior quadrant	48.94 ± 6.61	45.92 ± 6.25	0.069
Nasal quadrant	49.04 ± 6.19	43.6 ± 6.05	0.001
Inferior quadrant	48.19 ± 6.50	45.80 ± 6.70	0.157
Deep layer of the retina—thickness by sector			
Whole image	262.59 ± 20.94	263.06 ± 23.24	0.933
Superior hemisphere	312.93 ± 18.19	314.85 ± 20.07	0.692
Inferior hemisphere	286.14 ± 77.62	292 ± 73.32	0.760
Fovear	270.07 ± 32.51	280.26 ± 38.77	0.261
Parafovea	301.04 ± 19.25	296.24 ± 20.7	0.349
Temporal quadrant	309.83 ± 36.45	296.21 ± 44.11	0.185
Superior quadrant	313.07 ± 28.79	318.06 ± 29.36	0.499
Nasal quadrant	287.03 ± 39.56	286 ± 37.49	0.916
Inferior quadrant	316.38 ± 24.36	318.91 ± 25.77	0.690

Values are given as a number (percentage), mean (standard deviation), and median (5th–95th percentile). *p* < 0.05 indicates statistical significance.

**Table 3:** The interdependence of predictor variables in a multivariate regression model found statistically significant in subjects of the BEN group.

Dependent Variable	Independent Variable	B	95% CI		*p*-Value
			Lower Bound	Upper Bound	
Whole image deep	Smoking	4.35	0.16	8.54	0.048
	Diastolic blood pressure	0.29	0.10	0.47	0.004
	Systolic blood pressure	−0.13	−0.25	−0.01	0.036
Deep parafovea	Smoking	6.31	1.01	11.61	0.022
	Total cholesterol	2.46	0.05	4.87	0.046
Deep superior hemisphere	Creatinine clearance	−0.17	−0.29	−0.05	0.009
	Urinary creatinine	0.001	0.0002	0.0018	0.046
	Smoking	5.31	0.04	10.59	0.049
Deep inferior hemisphere	Antihypertensive therapy	−247.75	−382.97	−112.53	0.001
	Urinary creatinine	0.02	0.01	0.03	0.000
	Diabetes mellitus type 2	123.35	39.47	207.23	0.006
	Age	3.46	0.05	6.86	0.047
Deep temporal quadrant	Total cholesterol	2.56	0.30	4.82	0.028
Deep superior quadrant	Urinary creatinine	0.001	0.0005	0.0015	0.005
	Diastolic blood pressure	0.21	0.05	0.37	0.013
Deep nasal quadrant	Diastolic blood pressure	0.40	0.18	0.62	0.001
	Systolic blood pressure	−0.17	−0.30	−0.03	0.016

**Table 4:** The interdependence of predictor variables in a multivariate regression model found statistically significant in the CKD group.

Dependent Variable	Independent Variable	B	95% CI		*p*-Value
			Lower Bound	Upper Bound	
CFT	BMI	3.77	1.00	6.54	0.009
	Diastolic blood pressure	−1.27	−2.29	−0.25	0.016
	Total urinary protein	0.042	0.003	0.081	0.037
FAZs	Urinary creatinine	0.001	0.0006	0.0014	0.008
	BMI	−0.007	−0.012	−0.002	0.011
	Diuretic treatment	0.07	0.01	0.12	0.031
Whole image superficial	Age	0.21	0.04	0.38	0.017
	Urinary creatinine	0.001	0.0007	0.0013	0.025
Whole image deep	Total serum protein	−57.15	−80.08	−34.22	0.000
Superficial superior hemisphere	Age	0.34	0.16	0.53	0.001
	Diabetes mellitus type 2	4.68	0.15	9.21	0.043
Superficial inferior hemisphere	Total serum protein	21.65	0.22	43.09	0.048
Parafovea superficial	Creatinine clearance	−0.09	−0.16	−0.02	0.012
Superficial superior quadrant	Creatinine clearance	−0.11	−0.19	−0.03	0.008
Superficial nasal quadrant	Creatinine clearance	−0.08	−0.16	−0.001	0.048
Fovea deep	sCRP	0.09	0.05	0.13	0.000
	Creatinine clearance	0.57	0.29	0.85	0.000
	Diastolic blood pressure	−0.62	−1.05	−0.19	0.006

## Data Availability

Data are contained within the article.
